# PHYSICAL FUNCTION AND MITOCHONDRIAL BIOENERGETICS IN PEOPLE LIVING WITH HIV AND CANCER

**DOI:** 10.1093/geroni/igae098.3688

**Published:** 2024-12-31

**Authors:** Alice Ryan, Kathleen Griffith, Sausan Jaber

**Affiliations:** University of Maryland School of Medicine, Baltimore, Maryland, United States; GWU and Baltimore VAMC, Baltimore, Maryland, United States; Baltimore VAMC, Baltimore, Maryland, United States

## Abstract

**Purpose:**

To determine physical fitness and function, and potential mechanisms of frailty including bioenergetic health that may differ between older adults living with HIV (PLW) without and with a history of cancer (PLW+C).

**Methods:**

Participants (n=34, mean±SD, 64±6.6 years) underwent a cancer-specific geriatric assessment, physical function and fitness testing. In a subset (n=21, PLW n=10 and PLW+C n=11 lung, anus, colon, prostate), platelets were assayed for parameters of bioenergetic functions (Agilent Seahorse XFe96) in the presence of substrates for either glycolysis, the citric acid cycle, or fatty acid oxidation (FAO). Compounds were injected to determine basal, maximal, ATP-linked, and spare respiratory capacity.

**Results:**

PLW (100% male, 82% African American, BMI 28.7±6.3 kg/m2) enrolled. There were no differences between PLW (n=16) and PLW+C (n=18) in VO2peak (19.6±5.7 vs. 20.3±5.4 ml/kg/min), 6-minute walk distance (1400±385 vs.1273±415 ft), Short Physical Performance Battery score (10.8±1.5 vs.10.0±1.9), Berg Balance Scale score (52.2±3.1 vs 51.9±5.0), and Timed Up and Go (8.26±2.32 vs.10.2±5.3 s). Mito stress maximal (243.1±79.2 vs. 251.9±93.6 pmolO2/min/1x107cells) and spare respiratory capacity (113.8±61.5 vs. 121.3±51.4 pmolO2/min/1x107cells) are not significantly different between PLW and PLW+C; whereas FAO maximal (42.4±28.7 vs. 61.6±30.9 pmolO2/min/1x107cells) and spare respiratory capacity (30.6±25.0 vs. 56.9±29.5 pmolO2/min/1x107cells) tended to be higher in PLW+C. Conclusions: Although Mito Stress bioenergetic parameters of platelets from PLW+C or PLW are not different, the trend toward higher maximal and spare respiratory capacity in the presence of the FAO substrate in PLW+C may imply that their platelets have a higher reliance on FAO for fuel.

